# Application of microtechnologies for the vascularization of engineered tissues

**DOI:** 10.1186/2045-824X-3-24

**Published:** 2011-10-31

**Authors:** Robert Gauvin, Maxime Guillemette, Mehmet Dokmeci, Ali Khademhosseini

**Affiliations:** 1 Harvard-MIT Division of Health Sciences and Technology, Massachusetts Institute of Technology, Cambridge, MA, USA; 2 Center for Biomedical Engineering, Brigham and Women's Hospital, Harvard Medical School, Boston, MA, USA; 3 Wyss Institute for Biologically Inspired Engineering, Harvard University, Boston, MA, USA; 4 Defence Research and Development Canada (DRDC), Valcartier, QC, Canada

**Keywords:** microfabrication, biomimetic approaches, modular assembly

## Abstract

Recent advances in medicine and healthcare allow people to live longer, increasing the need for the number of organ transplants. However, the number of organ donors has not been able to meet the demand, resulting in an organ shortage. The field of tissue engineering has emerged to produce organs to overcome this limitation. While tissue engineering of connective tissues such as skin and blood vessels have currently reached clinical studies, more complex organs are still far away from commercial availability due to pending challenges with *in vitro *engineering of 3D tissues. One of the major limitations of engineering large tissue structures is cell death resulting from the inability of nutrients to diffuse across large distances inside a scaffold. This task, carried out by the vasculature inside the body, has largely been described as one of the foremost important challenges in engineering 3D tissues since it remains one of the key steps for both *in vitro *production of tissue engineered construct and the *in vivo *integration of a transplanted tissue. This short review highlights the important challenges for vascularization and control of the microcirculatory system within engineered tissues, with particular emphasis on the use of microfabrication approaches.

## Introduction

Progress in the development of large tissue-engineered organs has so far been limited by the inability to generate sophisticated three dimensional (3D) structures comprised of a functional vasculature. The vascular system is a dynamic environment comprised of a variety of cell types that constantly remodels itself under the influence of endothelial, immune, nervous and endocrine cells [1]. Vascular growth and remodeling are coupled with developmental and wound healing processes as well as the progression of various pathologies such as inflammation, cardiovascular diseases and cancer. Most of these processes depend on endothelial cells, which line the interior of blood vessels and form the endothelium. This interface between circulating blood and the surrounding tissues is responsible for proper solute transport and molecular exchange. It ensures the delivery of sufficient oxygen and nutrients to cells to maintain tissue homeostasis. Cells *in vivo *are found to be at most a few hundred microns away from the nearest capillary or blood vessel. Beyond this distance, diffusion is not effective and tends to reduce cell survival and function. Therefore, the inability to adequately vascularize engineered tissues results in inefficient transport of nutrients and metabolites and often leads to cell death and tissue necrosis. Moreover, vascularization of engineered tissues plays an important role in graft perfusion and is also crucial in facilitating the integration of the implanted material with the host vasculature [2,3].

**Figure 1:**
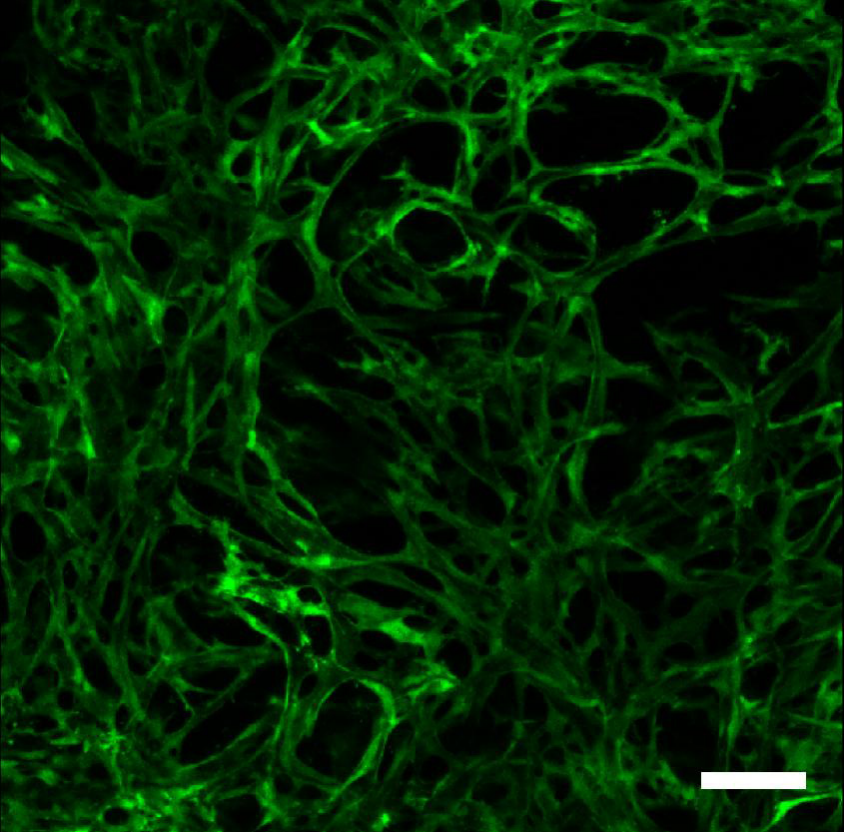
**Co-culture of fibroblasts and endothelial cells for one month in a collagen-GAG porous construct, resulting in a 3D capillary system within the biomaterial**. Confocal imaging of the full thickness of the scaffold showing green fluorescent protein (GFP)-labeled endothelial cells forming vascular channels throughout the 3D structure of the biomaterial.

The fabrication of tissue constructs often requires cell seeding of 3D scaffolds. These scaffolds are generally made of gels, foams or fibrous meshes and usually have basic macroscale properties that enable cell adhesion, migration and proliferation [4]. Although these properties are often sufficient to allow the formation of functional connective tissues such as skin [5,6], bladder [7] and cornea [8,9] and 3D tubular structures like blood vessels [10,11] and urinary tract [12], most tissue engineering approaches still lack the capability to sustain the growth of thick engineered organs [13]. Skin and cartilage have been among the first engineered tissues ready for clinical applications since they do not require extensive internal vasculature to survive *in vivo*. However, the fabrication of complex organs such as the heart or liver requires adequate vascular supply to ensure the survival of specialized cells within their 3D structure. To achieve this level of functionality, it is necessary to integrate a network of vessels ranging from a few microns to several millimeters within engineered tissues. The incorporation of this microcirculation into tissues and organs represents a considerable challenge which includes the engineering of vascular conduits having micron scale dimensions. It also requires a functional endothelium to improve vascular activity and avoid thrombosis, as well as other specialized cell types performing the physiological tissue function of interest. Various approaches have been proposed to design scaffolds comprised of a vascular network analogous to capillaries. These approaches rely on the release of growth factors [14,15] from the scaffolding material, the seeding of endothelial cells in the scaffold to promote angiogenesis [16] (Figure 1) or the use of microfabrication technologies to engineer branched microfluidic channels inside biocompatible materials [17]. Regardless of the application, these technologies all aim at improving mass and fluid transport as well as oxygen diffusion in engineered tissues produced *in vitro*.

### Microvascularization of Engineered Tissues through Angiogenesis and Inosculation

The need for adequate solute transport in cell-seeded scaffolds is essential for tissue survival and function. A key approach in attempting to induce the growth of a vascular network within 3D engineered tissue has been the incorporation of growth factors into the scaffolding material. It was shown that a macroporous scaffold, obtained by particle leaching, freeze drying or other pore forming technologies and functionalized with growth factors such as vascular endothelial growth factor (VEGF), basic fibroblast growth factor (bFGF) and platelet-derived growth factor (PDGF), can trigger the formation of vascular structures following *in vivo *implantation [18-22].

Both natural and synthetic scaffolding materials have been loaded with these pro-angiogenic molecules, leading to the sprouting of capillary beds within the constructs. However, the lack of directed growth of blood vessels to enable interconnectivity between the capillary networks still remains the biggest challenge of this technology.

Microvascular structures incorporated in tissue engineering scaffolds prior to their implantation can also be obtained by the co-culture of endothelial cells with the cell types of interest regarding tissue function. This cell-based approach uses the ability of endothelial cells to release growth factors and promotes the formation of capillaries *in vitro*. The resulting angiogenesis phenomenon can be explained by the remodeling that occurs within the construct, which is driven by the endothelial network that activates the release of pro-angiogenic factors in the construct. The remodeling of the extracellular matrix (ECM) allowing the formation of vasculature is driven by matrix-metalloproteases (MMPs), regulating cell proliferation and migration within the tissue [2]. Using a skin model, it has been originally demonstrated that a capillary network can be successfully produced by culturing human umbilical vein endothelial cells (HUVECs) into the dermal part of the engineered substitute leading to the formation of long-lasting and perfused blood vessel networks following *in vivo *transplantation [3,16,23-25]. This inosculation enabled the perfusion of the tissue engineered capillary network by the vasculature of the host following implantation. The cell-based approaches have led to many interesting results including vascularized muscle [26], cardiac tissue [27], bone [28] and blood vessel [29], all of which have been implanted in animal models and have shown improved perfusion and inosculation with the host vasculature. Similar results were obtained when HUVECs were cultured in a 3D collagen ECM *in vivo*, where a stable tree-like structure of a branched network was observed for an extended period of time [30]. Several other recent studies also suggested that mesenchymal stem cells and endothelial cells have the ability to interact together to form a stable vascular network of capillaries both under *in vitro *and *in vivo *conditions [31,32].

*In vivo *studies also demonstrated that the presence of vascular structures formed *in vitro *greatly accelerated inosculation of the implanted tissue with the host vasculature. Results from L'Heureux *et al*. have shown that the formation of a vasa vasorum in the wall of a tissue engineered blood vessel (TEBV) occurred 3 months following implantation *in vivo *[33].

Similarly, Guillemette *et al*. showed that a TEBV comprised of a vasa vasorum engineered *in vitro *(TEBVwVV) allowed for complete tissue integration and functional vasa vasorum activity after only 2 weeks *in vivo *[3]. These studies have provided evidence for the need to incorporate a capillary network into engineered tissues prior to implantation, showing improved and accelerated tissue integration and capillary-like structure formation which enabled connections with the host vasculature *in vivo*. Therefore, rapid formation of a vascular network between the engineered tissue and the host at the transplanted site, similar to the process observed during wound healing, represents a promising solution to provide implanted cells with adequate supply of oxygen and nutrients. However, this strategy does not provide a sustainable solution that can enable both perfusion *in vitro *and inosculation *in vivo*. In addition, these approaches did not result in the creation of organ scale constructs *in vitro*. In an attempt to engineer vasculature into engineered tissues and organs, microscale technologies and microfluidics systems have emerged as efficient tools to create easily perfusable channels in biocompatible and biodegradable 3D scaffolding materials.

### The use of Microtechnologies to Engineer Vascularization in vitro

The application of microtechnologies to biomaterials can be used to reproduce the capillary network and allow the flow of culture medium through a construct during *in vitro *studies. Unlike angiogenesis and inosculation approaches, microfabricated devices with integrated microvasculature can be optimized to provide a uniform distribution of flow and mass transfer across the scaffolding material and thus provide the cells with an adequate supply of nutrients.

Soft lithographic and micromolding processes have been used to create microfluidic devices consisting of branched networks that can be connected to perfusion systems *in vitro *[17]. These technologies have been applied to polymers such as poly(dimethyl siloxane) (PDMS), poly-lactic (co-glycolic acid) (PLGA) and polyglycerol sebacate (PGS) in which channel networks can be perfused and seeded with vascular cells [34-37]. These techniques have been shown to be useful to regulate the formation of vascular networks in a precise and efficient manner. The design of functional microvascular networks involves the integration of multiple parameters such as the geometry of branching and bifurcations, fluid mechanics, mass transport and structural rigidity.

These properties are of utmost importance since they greatly influence the stability, oxygen and nutrient distribution and therefore the functionality of engineered tissues. Microfluidic systems can effectively transport solutes in capillary channels ranging from a few millimeters down to micrometers [38]. The control of fluid mechanics and mass transport over this wide range of dimensions has been used to study bioactive molecules and therapeutics in cardiovascular research and tissue engineering [39]. Sophisticated devices have recently been fabricated to reproduce a lung assist apparatus allowing the blood being perfused within the microchannels to be oxygenated by flowing through many parallel capillary-like channels analogous to the native lung architecture [40]. Although optimization of the gas transfer membrane and characterization of the blood flow in the device are still needed, this is a good example demonstrating the potential of microfabrication technologies to generate vascularized platforms for tissue engineering. Similarly, it was shown that microvascular cells could be seeded in the device to form a confluent endothelium on the walls of the vascular channels [41]. These studies demonstrated that microfabricated devices comprised of a fluidic network modeled on human vasculature can be successfully inosculated *in vivo *[41].

Even though previous work has shown that microscale channels can be engineered *in vitro*, there is actually no available method to consecutively branch multi-dimensional channels inside a scaffold [42]. Top-down fabrication processes are inherently planar in nature and therefore 3D structures mostly result from stacked 2D structures which are comprised of channels having rectangular cross sections [43]. Moreover, most microfabrication techniques have been developed for materials that are unable to sustain cell encapsulation, which represents a limitation of this approach to generate large vascularized tissues [36,44,45]. Few attempts were made to engineer perfused microfluidic devices in which two different cell types can be cultured [36,46], but a vascularized tissue with parenchymal cells has yet to be created and there are still no methods to build sustainable large cell-laden structures with multi-dimensional branched networks. Other microscale fabrication approaches such as bioprinting and stereolithography are currently being investigated to create 3D branching vascular networks [47-50]. However, these methods not only require specialized facilities and expensive equipment, but the fabrication processes involved are usually time consuming.

### Biomimetic Approaches for Engineering Tissue Vascularization

Cells in the body are in contact with a complex 3D environment comprised of a combination of soluble factors as well as the ECM and basement membrane proteins found in the tissue in which they reside. Most tissues consist of multiple cell types organized into hierarchical structures that allow them to regulate their function. Modular tissue engineering, or bottom-up approaches, have recently emerged as powerful fabrication methods to generate 3D structures that mimic this organization and that recapitulate tissue structure and spatial resolution [51-54]. These techniques aim to generate biomimetic mesoscale structures by engineering microscale components and by using them as building blocks to fabricate larger tissue structures [55]. This approach has been used to control the cell microenvironment and the macroscale properties of relatively large and complex engineered tissues [56,57].

**Figure 2:**
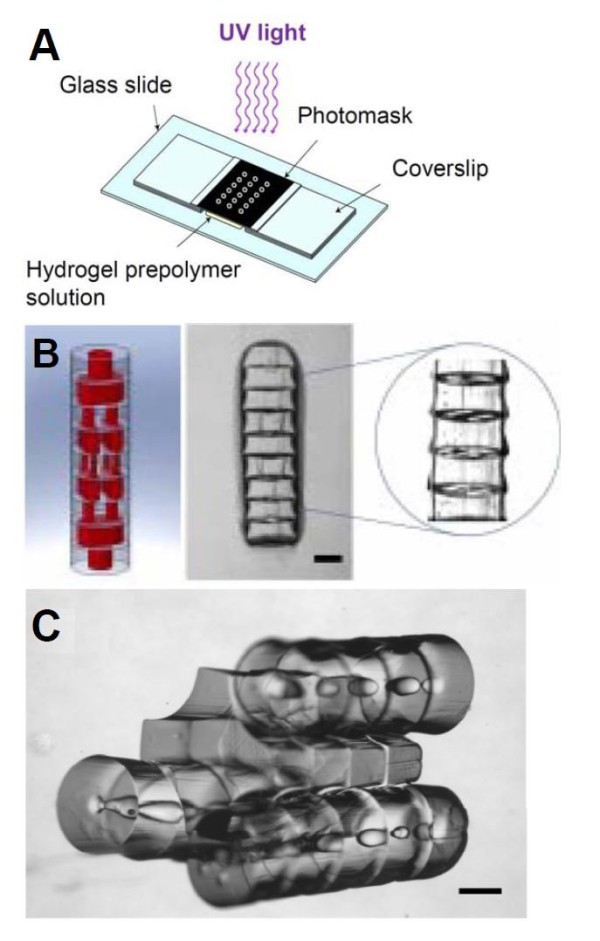
**Directed assembly of microgels using a modular approach**. Schematic representation of a high-throughput photolithographic method (A). Design image of microgel arrays assembled into tubular structures embedded with 3D branching lumens and actual phase image of the microgel assembly after secondary crosslinking (B). Phase image of microgels following a sequential and directed assembly process (C). Scale bar: 500 μm. (Adaptation from Du *et al*., Sequential assembly of cell-laden hydrogel constructs to engineer vascular-like microchannels, Biotechnol Bioeng, 2011, Copyright Wiley-VCH Verlag GmBH&Co. KGaA. Reproduced with permission.)

Microgels are microscale hydrogels fabricated by merging microscale fabrication and hydrogel chemistry. They exhibit properties similar to native ECM, can sustain cell encapsulation and have tunable geometrical, mechanical and biological properties which make them excellent candidates for tissue engineering applications. Based on these characteristics, cell-laden microgels were fabricated and then assembled into 3D tissue constructs to create precise

microarchitecture containing repeated functional units that mimic the *in vivo *structure of tissues and organs [52,54,58,59]. The directed assembly of microgels can be driven by simple thermodynamic processes in a two-phase oil-aqueous reactor [54,57]. Recent work has also shown the potential of modular assembly to produce vascularized tissues *in vitro *[60,61]. Arrays of microgels with precisely defined structures and channels have been produced by photolithography and were assembled in a controlled manner resulting in 3D structures with multidimensional interconnected lumens resembling native vasculature (Figure 2) [61]. The validation of this biofabrication process was assessed by placing endothelial and smooth muscle cells inside the microgels following a precise and concentric fashion mimicking the organization of blood vessels. The complete assembly was later strengthened by a secondary crosslinking step and it was shown that these assemblies could be perfused with fluids [61].

One of the challenges of directed self-assembly technology resides in the difficulty to scale-up the tissue produced. Larger structures have recently been built using lock-and-key principles and modular assembly [56]. Ongoing work is currently focusing on long-term perfusion aiming at developing mature and functional vasculature *in vitro*. Maintaining physiological functionality and blood flow in a high-density vascular network with optimized oxygen transfer conditions is critical to keep the tissue in an appropriate state of homeostasis. This biomimetic approach can also be utilized in the design of organ-on-a-chip technologies aiming at the fabrication of precise and reliable small scale models that can later be used for drug screening and physiological *in vitro *studies [62].

## Conclusions

One of the major limiting factors in the field of tissue engineering is the difficulty to generate functional 3D tissues due to the inability to integrate vascular structures into scaffolds. Building networks of vessels branched together into a complex interconnected structure connecting across multiple length scales remain one of the greatest challenges in tissue engineering. Most cells in the human body are within a few hundred microns from a capillary, allowing the delivery of adequate nutrients and supplies to the tissues and organs. Since most tissue engineering scaffolds are unable to provide such proximity for continuous solutes and oxygen flow, the engineering of large tissues severely lacks from diffusion and transport properties. The methods currently investigated to generate vasculature in scaffolds mainly involve the use of proangiogenic growth factors and cell-based approaches, which have shown promising results *in vivo*, but still cannot provide inlet and outlet vessels for *in vitro *perfusion. Despite all the advances in microfluidics, the use of microengineered 3D structures comprised of rationally designed and microfabricated channels offer limited functionality. These platforms do not provide a parenchymal space for cell types other than endothelial cells to grow within the constructs and present an integration problem with the host tissue. Modular and bottom-up approaches have recently emerged as promising biofabrication approaches in which functional microscale tissue building blocks can be assembled into 3D macroscale tissue constructs. These are relatively simple methods that allow the production of perfusable tissue, with precise control over the microscale features in a 3D construct. They are particularly promising in the case of organ engineering, where tissue requires perfusion and needs to perform a specialized physiological function. The precise design of microscale components in a high-throughput fashion combined with the capability to link these components together to generate larger structures represents a promising way to build vascularized 3D structures. Therefore, combining modular assembly methods with microfabrication technologies to engineer tissues and organs represent an effective method to control tissue architecture both at the micro and macroscale. This will be a major step forward in the field of tissue engineering that will not only result in the production of functional engineered tissues, but will also greatly help the translation of the technology from *in vitro *studies to *in vivo *applications.

## Competing interests

The authors declare that they have no competing interests.

## Authors' contributions

All the authors have been involved in the writing process and have read and approved the final version of the manuscript.
